# Evaluation of fronto-striatal networks during cognitive control in unmedicated patients with schizophrenia and the effect of antipsychotic medication

**DOI:** 10.1038/s41537-018-0051-y

**Published:** 2018-05-07

**Authors:** Elyse J. Cadena, David M. White, Nina V. Kraguljac, Meredith A. Reid, Adrienne C. Lahti

**Affiliations:** 1 Department of Psychiatry and Behavioral Neurobiology, University of Alabama at Birmingham, Birmingham, AL USA; 2 Department of Psychology, University of Alabama at Birmingham, Birmingham, AL USA; 3 Magnetic Imaging Research Center, Auburn University, Auburn, AL USA

## Abstract

To understand the mechanism of cognitive control dysfunction in schizophrenia, it is critical to characterize brain function without the confounding effect of medication. It is also important to establish the extent to which antipsychotic medication restores brain function and whether those changes are related to psychosis improvement. Twenty-two patients with schizophrenia, initially unmedicated and after a 6-week course of risperidone, and 20 healthy controls (HC) studied twice, 6 weeks apart, performed an fMRI task. We examined group and longitudinal differences in anterior cingulate cortex (ACC), striatum, and midbrain functional activity during performance of a Stroop color task as well as activity patterns associated with improvement in psychosis symptoms. Unmedicated patients showed reduced functional activity in the ACC, striatum, and midbrain compared to HC. Post hoc contrasts from significant group-by-time interactions indicated that, in patients, drug administration was associated with both activity increases and decreases. In unmedicated patients, greater baseline functional activity in the striatum and midbrain predicted subsequent better treatment response. Greater changes in functional activity in ACC and ventral putamen over the course of 6 weeks positively correlated with better treatment response. Unmedicated patients show reduced activity in brain networks pivotal for cognitive control and medication is associated with functional changes in these regions. These results suggest a mechanism by which antipsychotic medication has a beneficial effect on cognition. Our results also support the notion that treatment response is determined by a combination of the baseline pattern of brain function and by the pharmacological modulation of these regions.

## Functional imaging: Antipsychotic impact on brain activity

Functional imaging reveals brain regions altered in schizophrenia and how their pretreatment states can predict patient outcomes. As antipsychotic medications modify brain activity, a lack of studies on unmedicated patients makes it difficult to ascertain brain changes in schizophrenia itself or assess their clinical relevance. Adrienne Lahti, of the University of Alabama at Birmingham, and colleagues from the United States compared functional MRI scans of patients before and after 6 weeks of risperidone medication. Before treatment, they found significantly less activity, compared to healthy participants, in several brain areas during the performance of a stimulatory task. In unmedicated schizophrenia, a greater level of activity in certain brain networks correlated with positive treatment response, as did the levels of medication-mediated brain activity change. This study provides further evidence that medication response is partially determined by baseline neural function.

## Introduction

Dysfunction of fronto-striatal networks is widely reported in schizophrenia (SZ).^1–5^ Known abnormal dopaminergic transmission^6^ might contribute to these alterations, as both L-dopa administration^7^ and dopamine (DA) depletion^8^ are associated with changes in fronto-striatal functional connectivity. However, abnormalities cannot be definitively attributed to these pathophysiological alterations, since most studies in SZ have been conducted in medicated patients, and antipsychotic drugs (APDs) have prominent functional effects in these regions.^9–11^ It is therefore critical to characterize the extent of fronto-striatal dysfunction without the confounding effect of medication, which may also be relevant for other symptom domains such as cognition that are not improved with APDs. In addition, because fronto-striatal networks receive DA projections from the substantia nigra/ventral tegmental area (SN/VTA), these projections are likely to be critical for antipsychotic action.^12^ We have previously demonstrated the importance of the proper modulation of the ventral striatum and anterior cingulate cortex (ACC) to achieve good treatment response.^11^ Characterization of changes in these networks associated with treatment response could provide biomarkers to assist with the determination of drug effectiveness and help determine the basis of the considerable variability in treatment response.

Here we used a longitudinal design to evaluate brain function in initially unmedicated patients with SZ before and after a 6-week trial of APDs while controlling for the effect of time on the blood oxygen level-dependent (BOLD) signal in a group of healthy controls (HC) scanned 6 weeks apart. Our goals were to characterize (1) a network of regions including ACC, striatum, and SN (referred as cingulo-nigro-striatal network thereafter) in unmedicated SZ, (2) changes in this network induced by risperidone, a frequently used APD, and (3) cingulo-nigro-striatal BOLD patterns associated with treatment response. To engage this network, we used a Stroop task, a prototypical cognitive control task.^13^ Underscoring the relevance of this task to our goals, both striatal DA synthesis and DA receptor availability have been shown to correlate with cognitive control performance,^14,15^ including during Stroop performance, and a meta-analysis in Parkinson’s disease indicated that, of all the executive tasks surveyed, performance decrements on the Stroop were the largest.^16^

Based on prior findings,^17–19^ we hypothesized that we would observe reduced ACC BOLD signal as well as cingulo-nigro-striatal BOLD patterns that are predictive of subsequent good response to medication in unmedicated SZ. We also hypothesized that BOLD signal changes in the ventral striatum and the ACC^9,11,20^ would be correlated with treatment response.

## Results

### Demographics and Stroop behavior

HC and SZ did not differ in age, gender, parental socioeconomic status, or smoking (Table 1). Correct response reaction time (RT) showed a significant effect of group (*F*_1, 40_ = 4.47, *p* < 0.05), condition (*F*_1, 116_ = 122.88, *p* < 0.001), and a group×time interaction (*F*_1, 116_ = 4.00, *p* < 0.05). HC had faster congruent RT than unmedicated SZ (p < 0.05) and faster congruent and incongruent RT than medicated SZ (*p* < 0.05). There were no differences in RT between unmedicated and medicated SZ. No significant differences in error commission or missing trials were observed for group, time, or interactions (all *p* > 0.05; Table 1).

**Table 1:** Demographics, clinical, and behavioral measures

	SZ (*n* = 22)		HC (*n* *=* 20)		*t*/*x*^2^	*p*-Value
Age, years	33 (9.78)		33.05 (9.31)		−0.002	0.99
Sex, M/F	17/5		14/6		0.63	0.54
Parent SES^a^	7.89 (5.85)		5.68 (3.92)		1.34	0.19
Smoking status (smoker/non-smoker)	19/3		10/10			
Smoking, packs per day	0.73 (0.54)		0.39 (0.57)		1.97	0.06
Medication naive	*n* = 9					
Months off medication	27.75 (49.99)					
Diagnosis (schizophrenia/schizoaffective)	(19/3)					
Age of onset, years	21.857 (3.38)					
RBANS total^b^	70.55 (12.67)		93.5 (14.81)		−2.89	0.006
	SZ 0	SZ 6^c^	HC 0	HC 6		
BPRS^d^						
Total	48.59 (10.32)	29.52 (8.14)			8.88	< 0.001
Positive	8.86 (2.48)	4.52 (2.58)			7.82	< 0.001
Negative	7.05 (2.38)	5.14 (2.31)			3.01	0.007
Task reaction time, s						
Congruent	0.91 (0.18)	0.90 (0.19)	0.80 (0.10)	0.79 (0.13)		
Incongruent	1.04 (0.18)	1.03 (0.23)	1.00 (0.14)	0.91 (0.10)		
Stroop	0.13 (0.07)	0.13 (0.07)	0.19 (0.09)	0.14 (0.08)		
Missing trials						
Congruent	8.45 (11.86)	6.80 (13.76)	2.85 (8.38)	2.75 (5.30)		
Incongruent	4.32 (7.08)	4.20 (7.85)	1.05 (2.63)	1.25 (2.61)		
Task errors						
Congruent	10.71 (15.83)	8.06 (12.49)	2.75 (4.28)	4.30 (7.14)		
Incongruent	4.52 (5.23)	3.28 (3.75)	4.25 (5.46)	3.90 (5.09)		

Mean (SD) unless indicated otherwise

*SZ* schizophrenia, *HC* healthy control, *SZ 0* unmedicated baseline schizophrenia, *SZ 6* 6 weeks medicated schizophrenia, *HC 0* healthy controls baseline, *HC 6* healthy controls 6 weeks

^a^Ranks determined from Diagnostic Interview for Genetic Studies (1–18 scale); higher rank (lower numerical value) corresponds to higher socioeconomic status; data not available for 4 SZ subjects

^b^Repeatable Battery for Neuropsychological Status. Data not available for 5 SZ subjects

^c^*n* = 20.

^d^Brief Psychiatry Rating Scale (1–7 scale); positive (conceptual disorganization, hallucinatory behavior, and unusual thought content); negative (emotional withdrawal, motor retardation, and blunted affect); data not available for 1 SZ subject

### Unmedicated SZ compared to HC

**Fig. 1:**
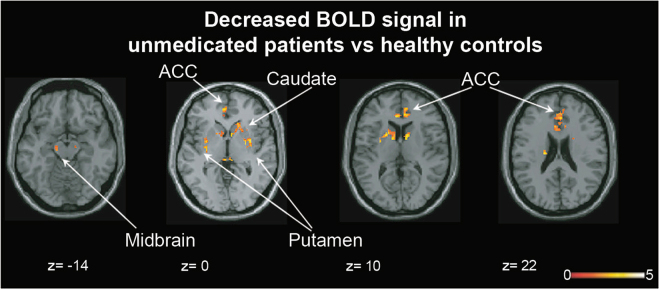
Between-group differences in BOLD activation during correct performance of the Stroop task. In unmedicated patients with schizophrenia, BOLD activity was decreased in midbrain, anterior cingulate cortex (ACC), bilateral caudate, and putamen compared to healthy controls (for details, see Table 2). Analyses were restricted to a mask encompassing the ACC, striatum, and midbrain using small-volume correction; *p* < 0.05, 47_SVC_. *z* coordinates refer to Montreal Neurological Institute (MNI) space. Regions labeled and indicated with arrows. ACC anterior cingulate cortex. Color bar on bottom indicates *t*-score

Compared to HC, unmedicated SZ displayed significantly less BOLD activity in the ACC, bilateral caudate, putamen, and midbrain during task performance (Fig. 1, Table 2).

**Table 2:** Significant regions in Stroop BOLD analyses

Region	Hemisphere	*x*, *y*, *z*	Voxels	Peak *t*-value
Between-group differences				
HC 0 > SZ 0				
Cluster 1		27, −3, 4	890	5.17
Putamen	R		459	
Caudate	R		383	
Cluster 2		−18, −16, 21	55	4.77
Caudate	L		52	
Cluster 3		−19, 9, 12	314	4.10
Caudate	L		230	
Putamen	L		69	
Cluster 4		15, −13, −1	52	3.96
Midbrain	R		52	
Cluster 5		−28, −1, 9	200	3.87
Putamen	L		188	
Pallidum	L		4	
Cluster 6		−4, 34, 10	614	3.64
ACC	IH		609	
Cluster 7		−10, −13, −3	261	3.54
Midbrain	L		261	
Cluster 8		6, −25, −7	201	3.53
Midbrain			196	
Lingual	R		17	
Cluster 9		−4, 45, 1	153	3.18
ACC	L		150	
Cluster 10		0, 19, 30	258	2.93
ACC	IH		254	
Cluster 11		9, −14, −21	122	3.04
Midbrain	R		119	
Full-factorial group×time interaction				
ACC	L	−6, 30, 14	84	
Putamen	R	28, 6, 3	53	
Caudate	R	12, 7, 11	29	
Midbrain	L	−4, −15, −10	141	
Thalamus	R	9, −30, 3	136	
Baseline BOLD associated with treatment response				
Cluster 1		15, 16, −4	65	3.96
Caudate	R		43	
Cluster 2		30, −17, 5	168	3.77
Putamen	R		163	
Cluster 3		−3, −23, −6	165	3.71
Midbrain	L		165	
Cluster 4		10, 7, 9	54	2.27
Caudate	R		48	
Change in BOLD associated with treatment response				
Cluster 1		31, −2, −6	79	5.06
Putamen	R		77	
Cluster 2		8, −15, −15	253	4.81
Midbrain	R		253	
Cluster 3		14, 30, 29	965	4.69
ACC	IH		943	
Cluster 4		22, 19, 11	56	2.96
Caudate	R		56	
Regions sharing overlapping BOLD response between contrasts				
Baseline between-group differences—full-factorial group×time interaction				
ACC	IH		48	
Baseline between-group differences—unmedicated schizophrenia associated with treatment response				
Putamen	R	27, −3, 5	72	
Caudate	R	20, 0, 15	53	

*x*, *y*, *z* refer to Montreal Neurological Institute coordinates. BOLD activation was striatum, ACC, and midbrain restricted (*p* < 0.05_SVC_).

*IH* inter-hemispheric, *L* left, *R* right

### Effect of risperidone

**Fig. 2:**
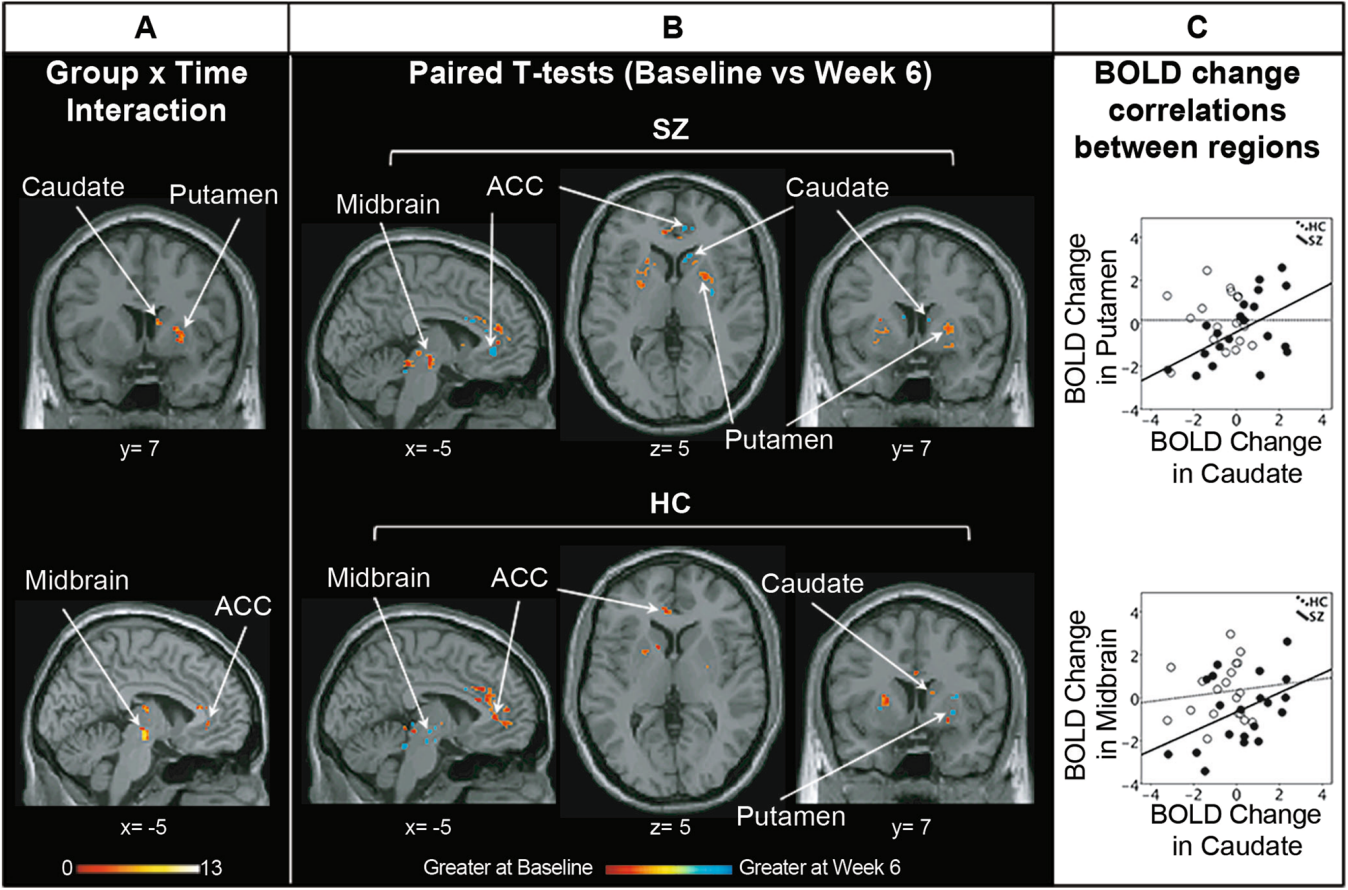
Effects of antipsychotic medication on BOLD activation. **a** BOLD full-factorial model (group×time interaction). Significant group×time interactions in BOLD activity were identified in the right caudate, right putamen, left ACC, and left midbrain (for details, see Table 2). Color bar on bottom indicates *F*-score. **b** Post hoc paired *T*-tests (baseline vs week 6) for each group (SZ: patients with schizophrenia; HC: healthy controls). Color bar on bottom indicates *t*-scores. Warm colors indicate a greater BOLD activity at baseline compared to week 6 and cold colors indicate the opposite. BOLD activity increased over the course of 6 weeks of risperidone in the right caudate and left ACC but decreased in the right putamen and left midbrain in SZ, with the opposite pattern observed in HC. All analyses were restricted to a mask encompassing the ACC, striatum, and midbrain using small-volume correction; *p* < 0.05, 29_SVC_. *x* and *y* coordinates refer to Montreal Neurological Institute (MNI) space. **c** In each group independently, changes in BOLD over the course of 6 weeks were correlated between each of the significant regions. In SZ, but not in HC, BOLD changes in caudate and in putamen as well as BOLD changes in caudate and midbrain were significantly correlated. Regions labeled and indicated with arrows. ACC anterior cingulate cortex

Significant group×time interactions were observed in the left ACC, right putamen, right caudate, and left midbrain (Fig. 2a, Table 2). Paired contrasts indicated that SZ BOLD significantly increased over the course of 6 weeks of risperidone in left ACC and right caudate but decreased in the right putamen and left midbrain. The opposite pattern was observed in HC (Fig. 2b, Table 2).

In SZ, but not in HC, BOLD changes in caudate and in putamen (*R*^2^ = 0.279, *p* = 0.017) as well as BOLD changes in caudate and midbrain (*R*^2^ = 0.190, *p* = 0.055) were significantly correlated (Fig. 2c). All correlations between regions identified in the group×time interaction are presented in Supplement Table 1.

### Treatment response

**Fig. 3:**
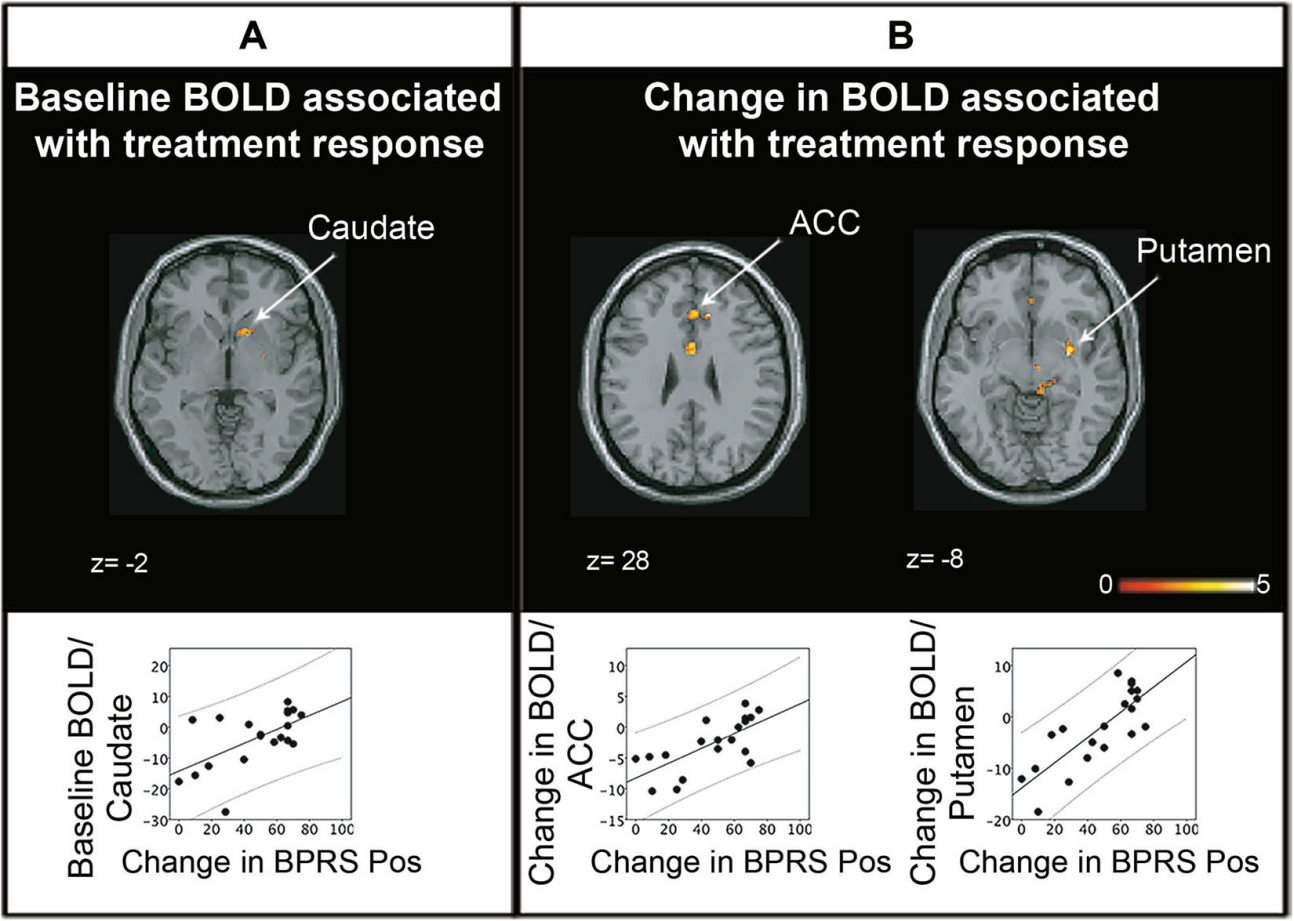
Associations between baseline BOLD (unmedicated) (**a**), changes in BOLD over 6 weeks and treatment response (**b**). In unmedicated patients, greater BOLD signal in the right caudate was predictive of subsequent better treatment response (improvement in BPRS Positive subscale score). Changes in BOLD in the ACC and right putamen over the course of 6 weeks were positively correlated with better treatment response (for details, see Table 2). Analyses were restricted to a mask encompassing the ACC, striatum, and midbrain using small-volume correction; *p* < 0.05, 49_SVC_. *z* coordinates refer to Montreal Neurological Institute (MNI) space. Color bar on bottom indicates *t*-score. BOLD activity values of significant regions for SZ subjects were plotted against treatment response. Solid lines indicate linear regressions and dashed lines indicate 95% confidence intervals. Regions labeled and indicated with arrows. ACC anterior cingulate cortex, BPRS Pos BPRS Positive subscale

In unmedicated SZ, greater BOLD activity in the right caudate, right putamen, and left midbrain was predictive of subsequent better treatment response (*p* < 0.05; Fig. 3a, Table 2). Additionally, greater changes in BOLD in the ACC, right ventral putamen, right caudate, and right midbrain were positively correlated with better treatment response (*p* < 0.05; Fig. 3b, Table 2). Using the response criterion of a 30% decrease in the Brief Psychiatric Rating Scale (BPRS) total score,^21–23^ 70% of patients in the study responded to treatment. Post hoc analyses on patient status found no significant difference between medication-naive and non-naive patients (*p* > 0.05).

## Discussion

This longitudinal study investigated a SN during performance of a cognitive task in unmedicated SZ and examined the effects of APDs on this network. In unmedicated SZ, we observed reduced BOLD activity in the ACC, caudate, putamen, and midbrain compared to HC. At baseline, greater task-induced BOLD activity in the striatum and midbrain was associated with subsequent better treatment response. Greater BOLD activity changes in the ACC, ventral putamen, and midbrain were also correlated with better treatment response.

### BOLD activity

Unmedicated SZ showed reduced BOLD activity in the midbrain, caudate/putamen, and ACC. Those results are consistent with the finding of reduced BOLD activity in prefrontal cortex seen during administration of the Continuous Performance Task in unmedicated patients compared to medicated patients and to HC.^19^ By simultaneously stimulating DA neurons and acquiring functional magnetic resonance imaging (fMRI) data, two optogenetic studies in rodents have recently established causality between phasic activation of SN/VTA DA neurons and BOLD activity changes in projection areas.^24,25^ Phasic firing of VTA DA neurons induced BOLD activity changes in VTA projections areas as well as in regions not receiving substantial VTA projections, suggesting that DA stimulation induced BOLD changes could be mediated directly by DA release or indirectly through multi-synaptic transmission,^25^ which is consistent with our observations. However, interpreting BOLD abnormalities in the context of potential DA dysfunction is challenging because the BOLD signal could reflect the integration over time of various patterns of DA firing (i.e., tonic, phasic) associated with task contingencies. Ongoing research is intensively investigating which pattern or combination of patterns is abnormal in SZ.^26,27^ Further work will need to establish causality between reduced task-induced BOLD signal and DA dysregulation in SZ. It is intriguing that we observed BOLD activity reductions in the midbrain as others have reported an increased midbrain BOLD activity during a working memory task, including in medication-naive first-episode patients.^28,29^

### Effects of antipsychotic medication

To disentangle the effect of medication and time, we conducted a group-by-time interaction on BOLD activity and observed significant interactions in the caudate, putamen, midbrain, and ACC. Post hoc contrasts indicated that, in SZ, drug administration was associated with both BOLD increases (ACC, caudate) and decreases (putamen, midbrain). Interestingly, while BOLD changes in those regions were not significantly correlated with each other in HC, some were in SZ, further supporting a drug-driven effect. Because of the prominent role of the ACC in cognitive control, these data suggest a mechanism by which antipsychotic medication has a beneficial effect, albeit limited, on cognition.^30^ Consistent with this, increased dorsolateral prefrontal cortex BOLD activation along with better behavioral performance during cognitive control was found in a group of medicated in contrast to a group of unmedicated patients.^19^ BOLD activity in both directions within the ACC in SZ supports the suggestion of functionally distinct ACC regions, with the dorsal area associated with motor, attention, and cognitive functions^31^ and the ventral area associated with emotion and autonomic functions.^32^ In a functional connectivity study, activity in the caudal ACC region was correlated with activity in sensorimotor circuits, while more rostral regions were associated with prefrontal region activity.^33^ Transition regions between rostral and caudal regions was also observed,^33^ suggesting integration or overlap of both types of processes. This may suggest more widespread ACC activity in unmedicated SZ in order to generate a sufficient response that becomes more regionally distinctive following medication.

The results also point to variability of BOLD response in HC over time, emphasizing the importance of accounting for time, but only a few studies have done this.^34^ Several factors could be driving this variability, such as habituation to task or scanner environment.

### Correlations with treatment response

Clinical response to APD is variable and currently unpredictable. Approximately 30% of patients will not improve with medications, and another 30% will show suboptimal response. There is a clear need for biomarkers to assist with the determination of drug effectiveness early in the course of treatment or before medication is initiated, such as in first-episode psychosis.

While there are some reports of relationship between baseline measures of brain structure and treatment response (see review in Dazzan et al. (2015)^35^), there have been limited findings on the relationship between patterns of brain activity with the likelihood of response to medication. Consistent with our prior findings,^17,36^ here we report that, *prior to treatment*, greater BOLD activity in the striatum and midbrain was associated with a greater chance of symptom improvement. In other words, prior to treatment, brain function is already arranged in a way that does or does not favor treatment response.

In addition, as psychosis improved, we observed BOLD activity changes in the midbrain, ventral putamen, and ACC. In a cohort of first-episode patients, Sarpal and colleagues reported a positive relationship between change in resting-state functional connectivity between the right dorsal caudate and ACC and improvement of psychosis.^3^ Putatively establishing a link between psychosis and cognitive control, in this study, we found that the greater the increase in ACC BOLD activity over the course of treatment during task performance, the greater the improvement in psychosis. These data replicate and extend our prior findings of regional cerebral blood flow changes in ACC that were correlated with good treatment response^11^ as well as the normalization with clozapine of an altered pattern of ACC activation seen during task performance.^20^ Others as well have reported changes in ACC/medial frontal cortex in association with APD treatment,^34,37–39^ underscoring the importance of the proper modulation of the ACC in order to reach adequate treatment response. APD treatment-related associations with the reverse Stroop effect (congruent > incongruent) are presented in Supplement Table 3. There was some degree of significant region overlap among the different contrast analyses. Bilateral ACC significantly overlapped in baseline group contrast and full-factorial interaction. Baseline group contrast also showed overlap with treatment response prediction at baseline in both the right caudate and putamen. It should be noted that none of the peak coordinates overlapped with BOLD change associated with treatment response.

### Strengths and limitations

To avoid confounding effects of medications and minimize variance in the data, we only enrolled unmedicated SZ, carefully matched groups on several key factors, and used a rigorous longitudinal design with a single antipsychotic medication. In addition, we partially controlled for the effect of time by scanning a group of HC 6 weeks apart. Symptom changes in the patients could have reflected placebo effects, compliance with treatment, and cannot automatically be entirely attributed to medication. As cognitive symptoms are not significantly affected by antipsychotic medication, potential changes in cognitive control may stem from alleviations in positive symptoms, improvements in attention,^40^ or practice effects.^41^ It should also be noted that the patients in this study may not be representative of all SZ patients, as they were able to provide consent for, tolerate scanning procedures, and perform a task. We used rigorous criteria to exclude subjects based on motion and found no significant group or time differences in head motion. Motivated by the nature of the task, we limited our study to the ACC regions of the prefrontal cortex. Owing to smoothing, we could not decidedly label midbrain subregions. However, all regions labeled as midbrain in our results fell within a mask restrictive to the SN and VTA.^42^

## Conclusion

In conclusion, in unmedicated patients, we found reduced BOLD activity in a SN during correct task performance. BOLD patterns that were predictive of good treatment response as well as changes in BOLD activity that were correlated with good treatment response were observed in this network as well. Our data support the notion that treatment response is determined by a combination of the baseline pattern of brain function as well as by the pharmacologic modulation of key regions, especially the ACC.

## Materials and methods

### Participants and study design

Twenty-eight subjects with SZ were recruited for this study from the psychiatry clinics and emergency room at the University of Alabama at Birmingham (UAB) based on being off antipsychotic medication for at least 10 days to ensure complete metabolism of any residual APDs and prevent any interactions. Twenty-five HC, matched on age, sex, smoking, and parental socioeconomic status, without personal or family history of psychiatric disorders in a first-degree relative were recruited using advertisements. Exclusion criteria were major medical or neurological conditions, substance use disorders (except for nicotine) within 6 months of imaging (drug screen was done prior to scanning), head injury with loss of consciousness >2 min, and pregnancy. Subjects gave written informed consent prior to participating in this UAB Institutional Review Board approved study. All SZ provided written informed consent and completed an Evaluation to Sign Consent Form.^43^

Diagnoses were established using subjects’ medical records and a consensus of two clinicians and then confirmed with the Diagnostic Interview for Genetic Studies.^44^ The Repeatable Battery for the Assessment of Neuropsychological Status characterized general cognitive function.^45^

SZ were scanned while unmedicated and after a 6-week trial with risperidone. Medication was managed by two psychiatrists (A.C.L. and N.V.K.), and dose determinations were based on therapeutic and side effects. Starting doses were 1–3 mg; titration was done in 1–2 mg increments. Use of concomitant medications was permitted as clinically indicated. Symptom severity was assessed weekly using the BPRS.^46^ Medication compliance was monitored by pill count at each visit. HC were scanned twice 6 weeks apart.

Subjects were excluded owing to excess movement (> 2-mm translation; 2° rotation within a run; 4 SZ, 2 HC) or lack of complete task performance (2 SZ baseline, 2 SZ week six, 3 HC), leaving 22 SZ and 20 HC at baseline and 20 SZ and 20 HC at week 6.

### Stroop task

Subjects performed a computerized version of the Stroop color-naming task.^47^ Stimuli consisted of three words: “RED”, “GREEN”, or “BLUE,” displayed in one of the corresponding colors. Trials were either “congruent” or “incongruent”, where the word and the color of the word differed in incongruent trials. Subjects were instructed to indicate the color but ignore the word and to respond as quickly and as accurately as possible. Responses were recorded by button press using an IFIS-SA system (In Vivo, Orlando, Florida) running E-Prime (version 1.2; Psychology Software Tools, Pittsburgh, PA). The event-related design consisted of three runs of 88 trials per run (~30% incongruent, 70% congruent). The 3-s trials were comprised of a word stimulus for 1.5 s and a fixation cross for 1.5 s. Participants completed a practice run before each scanning session.

### Image acquisition

Imaging was performed on a 3 T head-only MRI scanner (Magnetom Allegra, Siemens Medical Solutions, Erlangen, Germany), with a circularly polarized transmit/receive head coil. fMRI data were acquired using the gradient recalled echo-planar imaging sequence (repetition time/echo time [TR/TE] = 2100/30 ms, flip angle = 70°, field of view = 24 × 24 cm^2^, 64 × 64 matrix, 4 mm slice thickness, 1 mm gap, 26 axial slices). A high-resolution structural scan was acquired for anatomical reference (MPRAGE; TR/TE/inversion time [TI] = 2300/3.93/1100 ms, flip angle = 12°, 256 × 256 matrix, 1 mm^3^). Analyses between groups and across time found no significant differences in mean scan-to-scan head movement for the six movement parameters (See Supplement Table 2).

### Statistical analysis

Analyses were conducted in SPSS 20 (IBM SPSS Inc., Chicago, IL). Group comparisons were performed using chi-square or analysis of variance, as appropriate. Analyses of RT for correct trials [congruent, incongruent, and Stroop (incongruent–congruent)] and errors (congruent, incongruent) were analyzed using linear mixed models comparing fixed effects of group (HC vs SZ), time (unmedicated vs week 6), condition (congruent vs incongruent), and interactions. Post hoc analyses were performed where appropriate with Bonferroni correction.

### Image analyses

Data analyses were implemented in SPM8 (Wellcome Trust Centre for Neuroimaging). Preprocessing included slice-timing correction, realignment, reslicing at 1.5 mm isotropic voxels, motion/artifact correction using ArtRepair,^48^ DARTEL normalization, and smoothing (4 mm full-width at half-maximum Gaussian kernel). Analysis for the Stroop task consisted of a single-subject voxel-by-voxel general linear model. Five conditions were included: incongruent, congruent, stimulus repetitions (exact repetition of a previous trial^49^), error, and no response trials. The conditions were convolved with the canonical hemodynamic response function with temporal derivatives. The contrast of interest was correct incongruent trials minus correct congruent trials, subsequently referred to as the Stroop effect. A contrast *z*-map of the BOLD signal during the Stroop effect was generated for each participant at each time point.

Within SPM, we assessed between-group differences at baseline using a two-sample *t*-test. To examine the effect of APDs on BOLD, we employed a full-factorial analysis. Independent variables were included for group (HC vs SZ), time (unmedicated/baseline vs week six), and the interaction of group and time (group×time). We generated contrast images for the group×time interaction. To characterize these effects, paired-samples *t*-tests were conducted in both SZ and HC alongside the interaction (see Fig. 2, middle panel). We next tested the assumption that, in SZ but not in HC, *changes* in BOLD in those regions (where interactions were found) over the course of 6 weeks would be related to each other because of a drug effect. In each group independently, we correlated the *changes* in BOLD over the course of 6 weeks between each of the significant regions (i.e., for each group: BOLD change in caudate compared to BOLD change in putamen; BOLD change in caudate compared to BOLD change in midbrain, and BOLD change in caudate compared to BOLD change in ACC) (Fig. 2c). To conduct these analyses, contrast images were created using IMCalc (week 6–unmedicated/baseline) for each individual. Using REX (CIBSR Stanford University, CA), we extracted signal from these contrast images from the significant regions.

We used a regression analysis to determine whether BOLD signal at baseline in unmedicated SZ was related to subsequent treatment response and to determine whether the *change* in BOLD over the course of 6 weeks was related to treatment response. For the latter, contrast images were created using IMCalc (week six–unmedicated/baseline) and then entered into regression. To visualize the distribution of variance associated with these analyses, we extracted the first eigenvariate of the effect of interest in regions where a relationship with treatment response was observed and plotted the extracted values (*z*-scores) against treatment response. Treatment response was defined as the percentage of change on the BPRS psychosis subscale from baseline (*A*) to 6 weeks of risperidone (*B*): $\frac{{B - A}}{A} \times - 100.$

Analyses were corrected for multiple comparisons using small-volume-correction (SVC) in accordance with Gaussian random field theory (*p* < 0.05). In order to observe activity in the network of interest, results were restricted with a mask containing regions of the SN. The mask contained the ACC, putamen, and caudate from IBASPM 116 and midbrain from TD lobes as part of the WFU pickatlas.^50^ An image of the composed mask is provided in Supplement Fig. 1. To observe potential regional overlap amid analyses, significant regions from analyses (baseline group, group×time interaction, and predictors of symptom improvement) were combined into restrictive masks using IMCalc and then overlaid onto analyses not included in the composed mask. Significant overlapping regions are listed in Table 2. Positive and negative activation maps for incongruent, congruent, and Stroop effect conditions in both groups at both time points with SVC multiple comparison correction in the restricted SN are presented in Supplement Fig. 2. Whole-brain analyses in Supplement Fig. 3 are presented with multiple comparison correction using false discovery rate *p* < 0.05.

### Data availability

All the material will be available on request from the corresponding author. Clinical Trial registration number from ClinicalTrials.gov is NCT00937716.

## Electronic supplementary material

Supplementary Information
