# Supplementary material for: Evaluation of fronto-striatal networks during cognitive control in unmedicated patients with schizophrenia and the effect of antipsychotic medication
Source: NPJ Schizophr. 2018 May 7;4:8. doi: 10.1038/s41537-018-0051-y (PMC5938238; doi:10.1038/s41537-018-0051-y)
Supplement: Supplementary file 1 — Supplementary Information [file 41537_2018_51_MOESM1_ESM.docx]

| **Interaction Region Correlations** | **SZ** | | **HC** | |
| --- | --- | --- | --- | --- |
|  | **r** | **p** | **r** | **p** |
| ACC & Putamen | 0.08 | 0.75 | 0.32 | 0.17 |
| ACC & Caudate | 0.02 | 0.92 | 0.25 | 0.31 |
| ACC & Midbrain | -0.16 | 0.49 | -0.14 | 0.57 |
| Putamen & Caudate | 0.53 | 0.02 | 0.00 | 0.99 |
| Putamen & Midbrain | 0.11 | 0.66 | -0.12 | 0.61 |
| Caudate & Midbrain | 0.44 | 0.06 | 0.11 | 0.64 |

Supplement Table 1. Group x Time Interaction Region Correlations.

Abbreviations: SZ, schizophrenia; HC, healthy control. ACC, anterior cingulate cortex.

| **Motion Parameters** ^a^ | **SZ 0** | **SZ 6** | **HC 0** | **HC 6** |
| --- | --- | --- | --- | --- |
| X | -0.00 (0.02) | -0.02 (0.11) | 0.00 (0.01) | -0.00 (0.01) |
| Y | 0.01 (0.13) | 0.02 (0.09) | 0.02 (0.04) | -0.00 (0.03) |
| Z | 0.05 (0.04) | 0.06 (0.09) | 0.04 (0.04) | 0.04 (0.06) |
| Pitch | 0.00 (0.00) | 0.00 (0.00) | 0.00 (0.00) | -0.00 (0.00) |
| Roll | -0.00 (0.00) | -0.00 (0.00) | 0.00 (0.00) | -0.00 (0.00) |
| Yaw | -0.00 (0.00) | -0.00 (0.01) | -0.00 (0.00) | -0.00 (0.00) |

Supplement Table 2. Head Movement Parameters.

Abbreviations: SZ 0, unmedicated baseline schizophrenia; SZ 6, 6 weeks medicated schizophrenia. HC 0, healthy controls baseline; HC 6, healthy controls 6 weeks.

^a^ Mean (SD) for X, Y, Z. Mean degree (SD) for Pitch, Roll, Yaw.

Supplement Table 3. Congruent > Incongruent BOLD associated with treatment response.

| Region | Hemisphere | x, y, z | Voxels | Peak t-value |
| --- | --- | --- | --- | --- |
| **Congruent > Incongruent Baseline BOLD associated with treatment response** | | | | |
| Putamen | R | 24, 13, -7 | 64 | 3.93 |
| Midbrain | R | 12, -18, -13 | 74 | 3.55 |
| Midbrain | L | -4, -30, -9 | 51 | 2.50 |
| Caudate | L | -4, 7, 4 | 160 | 2.35 |
| ACC | R | 7, 40, 22 | 92 | 2.20 |
| Caudate | R | 10, 16, 3 | 100 | 2.06 |
| **Congruent > Incongruent Change in BOLD associated with treatment response** | | | | |
| Caudate | L | 10, 7, 12 | 71 | 3.87 |
| Midbrain | R | 8, -27, -4 | 188 | 3.80 |

Abbreviations: L, left; R, right.

x, y, z, refer to Montreal Neurological Institute coordinates. BOLD activation was striatum, ACC, and midbrain restricted (p<0.05_SVC_).


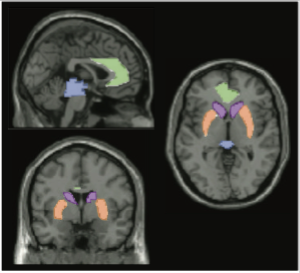


Supplement Figure 1. Inclusive mask of the cingulo-nigro-striatal network. Green, anterior cingulate cortex (ACC); purple, caudate; orange, putamen; blue, midbrain. Montreal Neurological Institute (MNI) coordinates (0, 0, 0).

Supplement Figure 2. Positive and negative main effects of congruent, incongruent, and Stroop condition. Analyses were restricted to a mask encompassing the ACC, striatum and midbrain using a small-volume correction; p<0.05. ACC: anterior cingulate cortex. Color bar on bottom indicates t-score.


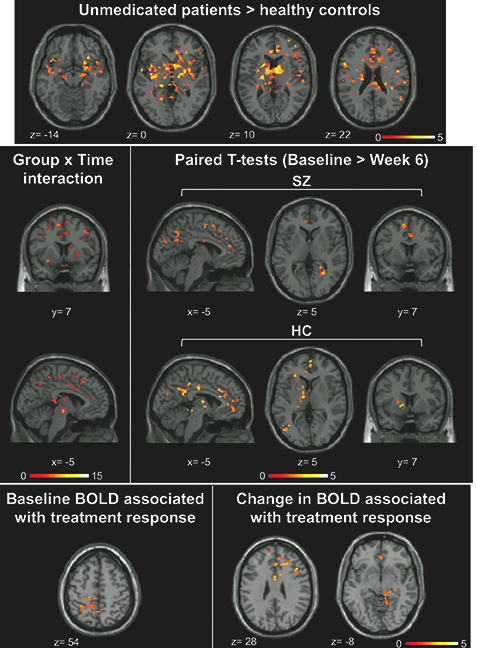


Supplement Figure 3. Whole brain results. Results were corrected with multiple comparisons; p<0.05, FDR. X, y, and z coordinates refer to Montreal Neurological Institute (MNI) space. Color bar indicates F-score in Group x time interaction, all else indicate t-score.
